# The Low Variability of Tc24 in *Trypanosoma cruzi* TcI as an Advantage for Chagas Disease Prophylaxis and Diagnosis in Mexico

**DOI:** 10.3390/pathogens12030368

**Published:** 2023-02-23

**Authors:** Ingeborg Becker, Haydee Miranda-Ortiz, Edith A. Fernández-Figueroa, Sokani Sánchez-Montes, Pablo Colunga-Salas, Estefanía Grostieta, Javier Juárez-Gabriel, Yokomi N. Lozano-Sardaneta, Minerva Arce-Fonseca, Olivia Rodríguez-Morales, Gabriela Meneses-Ruíz, Sergio Pastén-Sánchez, Irma López Martínez, Saúl González-Guzmán, Vladimir Paredes-Cervantes, Otacilio C. Moreira, Paula Finamore-Araujo, Julio C. Canseco-Méndez, Uriel Coquis-Navarrete, Laura Rengifo-Correa, Constantino González-Salazar, Myrna M. Alfaro-Cortés, Jorge A. Falcón-Lezama, Roberto Tapia-Conyer, Christopher R. Stephens

**Affiliations:** 1 Centro de Medicina Tropical, Unidad de Investigación en Medicina Experimental, Facultad de Medicina, Universidad Nacional Autónoma de México, Mexico City 04510, Mexico; 2 Unidad de Secuenciación, Instituto Nacional de Medicina Genómica, Mexico City 14610, Mexico; 3 Departamento de Genómica Poblacional, Instituto Nacional de Medicina Genómica, Mexico City 14610, Mexico; 4 Laboratorio de Diagnóstico, Facultad de Ciencias Biológicas y Agropecuarias Región Poza Rica-Tuxpan, Universidad Veracruzana, Tuxpan de Rodríguez Cano 92870, Mexico; 5 Instituto de Biotecnología y Ecología Aplicada, Universidad Veracruzana, Xalapa de Enríquez 91090, Mexico; 6 Department of Molecular Biology, National Institute of Cardiology “Ignacio Chávez”, Mexico City 14080, Mexico; 7 Departamento de Parasitología, Instituto de Diagnóstico y Referencia Epidemiológicos, Secretaría de Salud, Mexico City 01480, Mexico; 8 Laboratorio del Banco Central de Sangre del Centro Médico Nacional “La Raza”, Instituto Mexicano del Seguro Social, Mexico City 02990, Mexico; 9 Departamento de Investigación, Hospital Regional de Alta Especialidad de Zumpango, Zumpango 55600, Mexico; 10 Unidad de Investigación Médica en Inmunología e Infectología, Hospital de Infectología, Centro Médico Nacional “La Raza”, Instituto Mexicano del Seguro Social, Mexico City 02990, Mexico; 11 Laboratorio de Biología Molecular e Doencas Endêmicas, Instituto Oswaldo Cruz, Fiocruz 21040900, RJ, Brazil; 12 Centro de Ciencias de la Complejidad, Universidad Nacional Autónoma de México, Mexico City 04510, Mexico; 13 Fundación Carlos Slim, Mexico City 11529, Mexico; 14 División Académica de Ciencias de la Salud, Universidad Juárez Autónoma de Tabasco, Villahermosa 86100, Mexico; 15 Facultad de Medicina, Universidad Nacional Autónoma de México, Mexico City 04510, Mexico

**Keywords:** Chagas disease, Tc24, population genetics, DTUs, molecular epidemiology

## Abstract

(1) Background: Chagas disease is the main neglected tropical disease in America. It is estimated that around 6 million people are currently infected with the parasite in Latin America, and 25 million live in endemic areas with active transmission. The disease causes an estimated economic loss of USD 24 billion dollars annually, with a loss of 75,200 working years per year of life; it is responsible for around ~12,000 deaths annually. Although Mexico is an endemic country that recorded 10,186 new cases of Chagas disease during the period of 1990–2017, few studies have evaluated the genetic diversity of genes that could be involved in the prophylaxis and/or diagnosis of the parasite. One of the possible candidates proposed as a vaccine target is the 24 kDa trypomastigote excretory–secretory protein, Tc24, whose protection is linked to the stimulation of *T. cruzi*-specific CD8^+^ immune responses. (2) Methods: The aim of the present study was to evaluate the fine-scale genetic diversity and structure of Tc24 in *T. cruzi* isolates from Mexico, and to compare them with other populations reported in the Americas with the aim to reconsider the potential role of Tc24 as a key candidate for the prophylaxis and improvement of the diagnosis of Chagas disease in Mexico. (3) Results: Of the 25 Mexican isolates analysed, 48% (12) were recovered from humans and 24% (6) recovered from *Triatoma barberi* and *Triatoma dimidiata*. Phylogenetic inferences revealed a polytomy in the *T. cruzi* clade with two defined subgroups, one formed by all sequences of the DTU I and the other formed by DTU II–VI; both subgroups had high branch support. Genetic population analysis detected a single (monomorphic) haplotype of TcI throughout the entire distribution across both Mexico and South America. This information was supported by Nei’s pairwise distances, where the sequences of TcI showed no genetic differences. (4) Conclusions: Given that both previous studies and the findings of the present work confirmed that TcI is the only genotype detected from human isolates obtained from various states of Mexico, and that there is no significant genetic variability in any of them, it is possible to propose the development of in silico strategies for the production of antigens that optimise the diagnosis of Chagas disease, such as quantitative ELISA methods that use this region of Tc24.

## 1. Introduction

Chagas disease is considered the principal neglected tropical disease on the American continent. Both its incidence and geographical distribution have increased alarmingly in the last two decades. The disease is caused by a single species of haemoparasitic flagellate, *Trypanosoma cruzi*, which is transmitted by several species of blood-sucking bugs of the Triatominae subfamily. The modification of wild ecosystems, the increase in urbanisation, and demographic changes due to migration are all key factors for the dissemination of the disease. The life cycle of the parasite includes a wide range of mammalian hosts, including humans, in which it generates a wide range of manifestations, including heart disease, and digestive and/or neurological disorders. It is estimated that around 6 million people are currently infected with the parasite in Latin America [1], and 25 million live in endemic areas with active transmission [2]. The disease causes an estimated economic loss of USD 24 billion dollars annually, with a loss of 75,200 working years per year, and it is responsible for around ~12,000 deaths annually [1,3]. Additionally, blood transfusions and organ transplants or congenital transmission constitute new routes that have allowed for its dissemination to countries of the European Union and Southeast Asia [4,5].

Over the last 30 years, it has been assumed that the clinical manifestations of the disease depend on factors that are associated with the involved vector and the geographical region where the transmission occurred. For this reason, strategies have been implemented that identify the genetic diversity of the parasite, and associate it with virulence and disease progression. Likewise, it has been postulated that developing a better understanding of the genetic variability of the parasite is a fundamental factor in increasing the sensitivity of current diagnostic methods and supporting the possible development of a candidate vaccine.

This parasite has a clonal reproductive pattern that results in low genetic variability. Consequently, multiple strategies have been proposed for its classification, among which the use of discrete typing units (DTUs) stands out. This has allowed for the identification of six DTUs that are present in both animals and humans, where the most recent one, TcBat, presumably only infects bats [6]. The epidemiology of these DTUs is such that TcI predominates in human patients with Chagas cardiomyopathy in North America, while TcII–TcVI is widely associated with cardiomyopathy, megaoesophagus, and megacolon in South America [7,8].

According to the Ministry of Health of Mexico and the World Health Organisation (WHO), the country reported 10,186 new cases of Chagas disease during the period of 1990–2017 [9]. Nevertheless, the reliability of these numbers could be sensitive to the implemented diagnostic techniques and parasitic diversity. The international agency (WHO) estimates that more than one million people were infected with *T. cruzi* in 2006 across the country. The diagnostic algorithm of Chagas in Mexico, according to the Mexican Official Norm NOM-032-SSA2-2014, depends on the phase of the disease, and includes serological tests and PCR [10].

There have been multiple studies of the vectors of *T. cruzi* in Mexico, where it is estimated that there are around 33 species of triatomines, of which 27 were found infected with *T. cruzi* [11,12,13]. The presence of *T. cruzi* in humans and pets, and their exposure to it were reported both via serological tests and molecular biology methods, including quantitative (q-PCR) and conventional (c-PCR) polymerase chain reaction [14,15,16,17].

**Figure 1:**
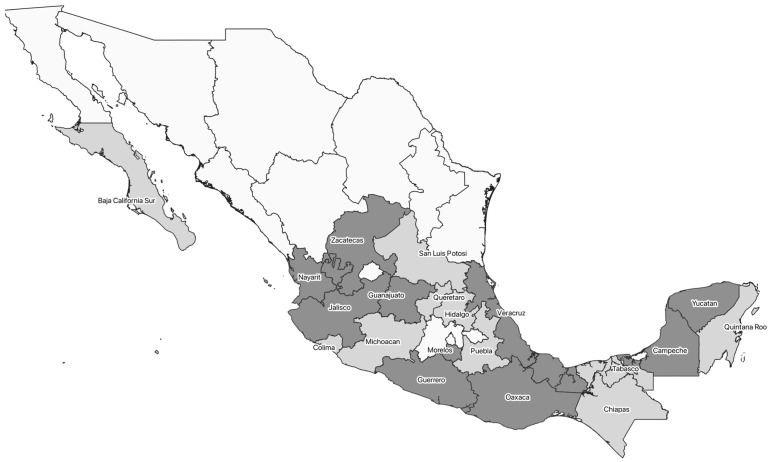
Distribution of all *Trypanosoma cruzi* DTUs in Mexico. Light grey represents the Mexican states where TcI had been previously recorded. Dark grey represents the Mexican states from which *T. cruzi* isolates were obtained for this study.

However, despite the high number of human cases of Chagas disease and the high richness of vectors reported across the entire country, only 15 studies have been carried out to identify circulating DTUs, with the confirmation of the presence of 6 of the 7 DTUs in 21 hosts, and 8 vectors from 20 of the 32 states (Table 1 and Table 2, and Figure 1). Furthermore, studies related to the typification of human cases of Chagas disease are even scarcer, demonstrating mostly the presence of TcI in human patients (Table 2).

Due to the public health importance of Chagas disease, multiple drugs and vaccines were proposed as treatments and/or prophylactic methods [33]. One of the possible vaccine candidates proposed is the 24kDa trypomastigote excretory–secretory protein, Tc24, whose protection is linked to the stimulation of *T. cruzi*-specific CD8^+^ immune responses [34,35,36].

Arnal et al. [37] evaluated the genetic diversity of this protein using TcI isolates from Central and South America. In their study, they found no genetic diversity in the entire sequence obtained by the whole-genome sequencing of the Tc24 protein. Additionally, they reported that, according to the conserved epitope of predicted CD8^+^ T cells, this protein is under a strong purifying selection model, rendering it an excellent vaccine candidate [34,35,36]. However, in this study, only strain H1b was used as a representative of the entire Mexican territory, leaving out information on uncharacterised Mexican *T. cruzi* isolates.

On the other hand, Mexican isolates of *T. cruzi* exhibit different degrees of virulence that, in a murine experimental infection model, showed diverse humoral and cellular immune responses [38]. Additionally, the genetic diversity of Mexican isolates via isoenzymatic analysis demonstrates subtle genetic variation among the southern sequences [39]. This information is suggestive of a certain level of diversification within TcI in Mexico. For this reason, the aim of the present study was to evaluate the fine-scale genetic diversity and structure of Tc24 in *T. cruzi* isolates from Mexico and to compare it with those populations reported in the Americas, with the aim to reconsider the potential role of Tc24 as a key candidate for the prophylaxis and improvement of the diagnosis of Chagas disease in Mexico.

## 2. Materials and Methods

### 2.1. Selection, Maintenance, and Proliferation of Isolates

As a part of an ongoing multidisciplinary and interinstitutional project to identify the ecoepidemiology of Chagas disease in Mexico, we typified the *T. cruzi* strains isolated from patients, nonhuman hosts, and vectors from two reference institutions, the Ignacio Chávez Institute of Cardiology and the Institute of Epidemiological Diagnosis and Reference from the National Institutes of Health. Additional TC24 sequences were taken from GenBank (Table 3).

Only parasites isolated from Mexico were grown in an LIT medium (Difco) supplemented with inactivated fetal bovine serum (SBF) at 27 °C for 7 days. The total obtained culture volume was centrifuged at 3200× *g* for 10 min, and the pellet was washed with PBS to clean and isolate the parasites. Subsequently, DNA extraction was performed using a High Pure PCR Template Preparation kit (Roche, Mannheim, Germany) following the manufacturer’s recommended procedures for DNA isolation from tissues. Samples were eluted with 100 µL of elution buffer. We performed the molecular typing of the strains by using multilocus sequence typing (MLST) for the identification of the DTUs [40]. As a panel of positive controls, we used *T. cruzi* DNA from subpopulations classified as DTUs TcI to TcVI (clones/strains: Dm28c (TcI), Y (TcII), INPA 3663 (TcIII), INPA 4167 (TcIV), LL014 (TcV) and CL Brener (TcVI)), donated from Otacilio C. Moreira’s personal collection.

### 2.2. Development of Primers

CodonCode Aligner v.9.0 software was used to generate primers for Tc24 gene amplification on the basis of the *T. cruzi* (Bug2148) reference genome (GenBank accession number NMZN00000000.1). Forward primer TC24F (5′-CAAGGAAGCGTGGGAGCG-3′) and reverse primer TC24R (5′-CAGCAAACTCGTCGAACGTC-3′) were used to generate a 490 bp amplicon using 12.5 µL QIAGEN Master mix (QIAGEN Inc., Hilden, Germany), 1 µL (10 µM) of each primer, 1 µL (1 ng) of extracted DNA, and 9.5 µL of bidistilled sterile water into a final volume of 50 µL. Additionally, bidistilled sterile water was used as negative control. The temperature conditions for the polymerase chain reaction (PCR) were as follows: 94 °C for 3 min, 32 cycles of 94 °C for 30 s, 59 °C for 30 s, 72 °C for 1 min, and 72 °C for 10 min; verification was performed via electrophoresis on a 1% agarose gel stained with Smartglow. Sequencing was performed for the samples with bidirectional sequencing in a 3730 × L DNA Analyzer.

### 2.3. Phylogenetic Analysis

ABI files were extracted to be analysed. Sequence data were edited, and global alignments were performed using the Clustal W algorithm in Mega 10.0. Sequences generated in this study were submitted to GenBank using the Bankit tool. To corroborate the DTU identity of each isolate, phylogeny using the TC24 region was assessed in two ways, as had previously been described for other vectors and pathogens [41,42]. The first approach was evaluated in IQ-TREE [43], evaluating the best substitution model with the ModelFinder algorithm [44], with a full-tree search for all available models according to the Bayesian information criterion (BIC). The maximum likelihood hypothesis was estimated using the best previously calculated substitution model; the branch support was evaluated with 10,000 ultrafast bootstraps and 10,000 additional replicates for the SH-aLRT branch test, according to the authors [43].

The second approach was using MrBayes 3.2 for Bayesian inference analysis [45]. To select the best substitution and partition model, we used PartitionFinder 2 with mrbayes models and the greedy scheme search, also considering the BIC [46]. This analysis was performed using the Markov chain Monte Carlo (MCMC) algorithm, and the best previously calculated substitution and partition model. A total of 3 hot and 1 cold chains in 2 independent runs of 10 million generations, sampling every 1000 generations, were used [47]. The final topology of the phylogenetic analysis was obtained via a majority consensus tree with a burn-in of 25%. Convergence and good sampling (ESS > 200) were evaluated in Tracer 1.7.1 [48].

### 2.4. Genetic Analysis

To ascertain the genetic diversity, we calculated the number of haplotypes, unique haplotypes, mutations, segregating sites, and unique sites, and haplotypic and nucleotide diversity in DNAsp 5.10 [49]. To identify the relationship among haplotypes, minimal union networks were constructed using the PopArt programme. Lastly, to evaluate the fine-scale genetic diversity, Nei’s genetic distances were calculated, but only among the sequences belonging to the TcI clade, using the adegenet R package [50] and considering the previously calculated K2P substitution model with the ModelFinder algorithm.

## 3. Results

### 3.1. Identification of the Isolates

Of the 25 analysed Mexican isolates, 12 (48%) were recovered from humans, followed by 6 (24%) recovered from unidentified triatomines, and 5 from identified ones—*Triatoma dimidiata* (3) and *Triatoma barberi* (2) (Table 3). Regarding the geographical distribution, the isolates came from 11 (33%) states of the Mexican Republic. The most isolates came from the state of Oaxaca, with 16% (4), followed by Campeche, Guanajuato, Morelos, and Yucatan with 12% (3) each. The states of Jalisco, Nayarit, Veracruz, and Zacatecas contained the lowest percentages, with just 4% (1) each. For one isolate, we had no data on the host or state of the republic from which it was recovered.

### 3.2. Phylogenetic Analysis

**Figure 2:**
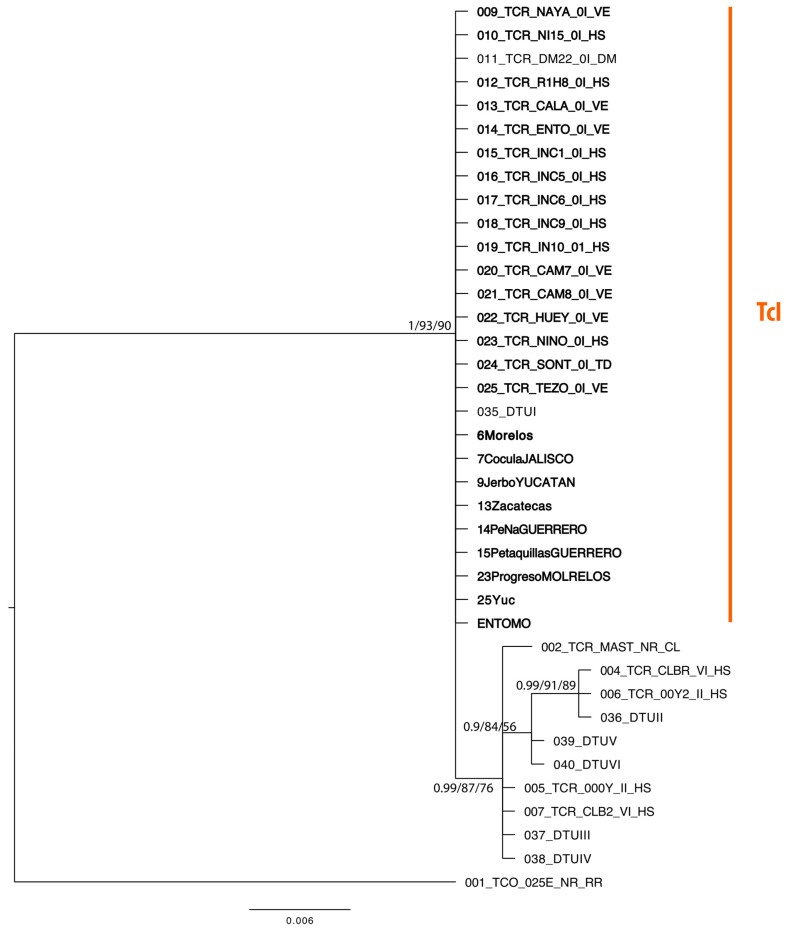
Bayesian inference phylogeny for Trypanosoma cruzi DTU I–VI based on 427 bp from the TC24 region. Branch labels show the branch support at each node according to posterior probabilities/SH-aLRT/boostrap from BI and ML analyses. Sequences in bold correspond to those obtained in this study. Bar scale represents nucleotide substitutions.

The best substitution model for ML analysis was K2P (BIC = 1772.224); for the BI, the best partition model was the HKY for the first and third positions, and F81 for the second position (BIC = 1969.107). In both analyses, the same general topology was recovered. However, the branch support was higher in the BI hypothesis. Both phylogenetic inferences revealed a polytomy in the *T. cruzi* clade with two defined subgroups. One subgroup formed by all sequences of the DTU I, and the second subgroup was formed by DTU II–VI, with both subgroups having high branch support (Figure 2).

### 3.3. Genetic Analysis

For the population genetic analysis, we constructed an alignment with 37 sequences of *T. cruzi* (6 available in GenBank and 31 recovered in this study (25 Mexican isolates and the 6 reference DTUs)). The final alignment consisted of 427 base pairs, with 421 conserved and 6 variable sites, 1 singleton (in position 150), and 5 parsimony informative sites (in positions 57, 82, 164, 124, 153, 325). We detected the presence of five haplotypes. The most frequently detected haplotype was H3, with 27 sequences (72.9%), followed by haplotypes H1 with 4 (10.8%) and H2 with 3 sequences (8.1%). Haplotype H2 was the most widely detected, occurring in four of the six DTUs (TcII, TcIV–TcVI), whereas H3 and H4 were only detected in two DTUs (TcII/TcVI and TcV/TcVI, respectively). The least frequent haplotype was H1, which was recorded once (Table 1). Haplotypic diversity (Hd) was 0.458, and nucleotide diversity was 0.00331.

**Figure 3:**
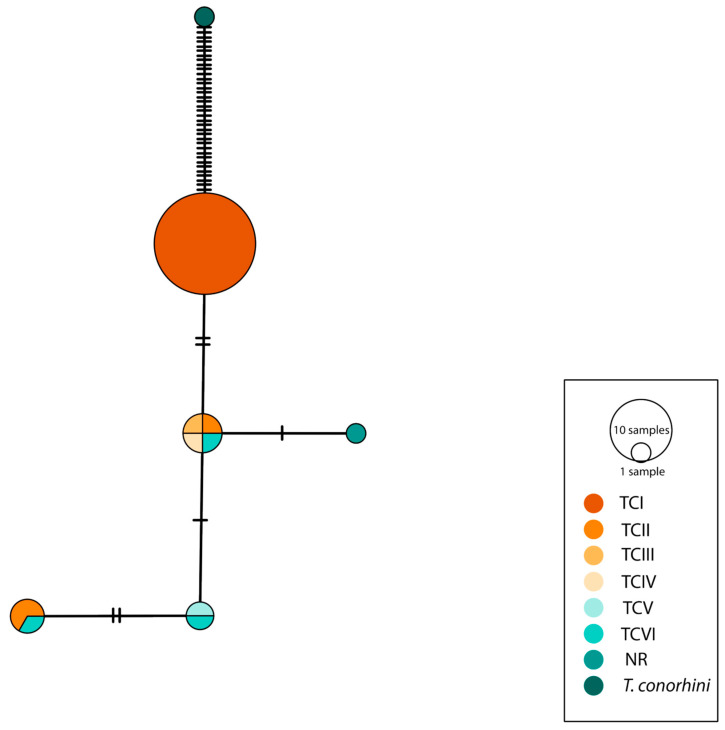
Haplotypic network for the TC24 fragment for DTU I–VI of *Trypanosoma cruzi*. Colours correspond to each DTU and the outgroup, *Trypanosoma conorhini*. NR: DTU not referred. Black lines represent the mutational steps between each haplotype.

**Figure 4:**
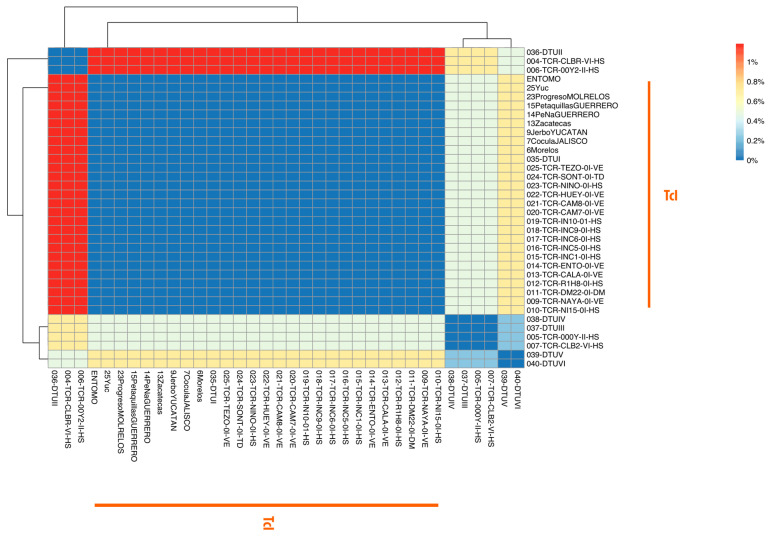
Heatmap of Nei’s genetic distances for the TC24 fragment for DTU I–VI of *Trypanosoma cruzi*. Colour gradient represents the percentage of differentiation when comparing the 37 sequences according to the scale bar on the right.

Minimal union network analysis revealed a separation of populations by DTU, with an overlap of several haplotypes among DTUs II–VI. Notably, haplotype 1 exhibited a single (monomorphic) haplotype throughout the entire distribution across Mexico and South America (Figure 3). This information was supported with Nei’s pairwise distances, where no sequences of DTU I exhibited genetic differences, but with approximately 10% of difference when DTUs II, III, and VI were compared to DTU I, and differences ranging from 6 to 8% compared with the sequences of DTU II–VI (Figure 4).

## 4. Discussion

Chagas disease constitutes an increasingly important public health problem in the American continent. Difficulties related to early diagnosis and timely treatment reduce the quality of life of infected patients. For this reason, the identification of targets that increase the sensitivity of the diagnosis and that can be used as vaccine candidates is imperative [33,34,35,36].

For this reason, the findings of the present study strengthen the possibility of using Tc24 as a molecule that can be implemented as a candidate for prophylaxis and therapy against this disease. In the present work, we analysed isolates from humans and vectors from several states of Mexico. The results show that, during the period of 1970–2005, the recovered strains corresponded to TcI. Population genetic analysis shows that there was no genetic diversity in this fragment in any of the Mexican TcI isolates or those recovered from Central America, Peru, and Brazil. A previous study demonstrated that this protein is under negative selection pressure, probably due to the relevant role of this molecule in flagellar production, and adhesion to the vector and potentially to the vertebrate host [37]. Historically, only the presence of infected human TcI was demonstrated in Mexico, although the presence of the six DTUs has been identified in various species of terrestrial vertebrates and triatomines found across the country. However, a recently conducted study on pregnant women from the southern state of Yucatan, Mexico demonstrated a high frequency of TcII, TcV, and TcVI DTUs (non-TcI DTUs), including mixed infections with TcI. Despite these novel findings, the presence of an infection by multiple DTUs in humans has been confirmed exclusively in the Yucatan Peninsula, in the southeast of the country, for which larger samplings are required in other regions to corroborate the actual prevalence and magnitude of the phenomenon [32]. For this reason, and in accordance with most of the demonstrated evidence, TcI is the most frequently reported DTU in humans in the country up to now.

Previous studies related to the analysis of the genetic diversity of other neglected tropical parasites have provided important information as to how more effective transmission mitigation or control strategies may be developed. The identification of the genetic variants that circulate in a region is essential in order to establish the potential efficiency of prophylactic vaccines. This was demonstrated in the currently used malaria vaccine against *Plasmodium falciparum* in Sudan and other African countries [51]. On the other hand, previous studies related to the evaluation of the genetic diversity of other neglected tropical parasites have provided relevant information about the strategies related to the mitigation of transmission or the effectiveness of the control, as in the case of *Plasmodium vivax* in the southeast of Mexico [52].

In this vein, our study reveals the presence of a highly conserved fragment of the Tc24 protein across TcI isolates from Mexico and from other countries in Latin America, thus providing a potential target for the development of a regional vaccine that could protect most of the people in risk areas where TcI is the main circulating genotype. Tc24 was postulated as a vaccine candidate, and was tested on murine [53,54], canine [55], and nonhuman primate [56] models, showing activity in decreasing the proliferation of parasites in the blood and decreasing cardiac damage in immunised animals.

Similarly, the analysis of genetic variants that circulate in populations of parasites can also improve the capacity for regional diagnosis, as serological diagnostic methods increase their sensitivity by establishing local or regional isolates as antigens [57]. This is relevant for Chagas disease, in that there is a problem in the serological diagnosis of this disease in both South and North America [58,59]. Commercial kits have been developed with total lysates of several DTUs from South America, mainly TcVI; therefore, sensitivity may decrease when screening sera from human patients infected with other DTUs [60,61]. On the other hand, an overestimation of cases in some regions is also possible, since some populations could be exposed to different parasitic variants or even other trypanosomatid parasites, such as *Leishmania*, and present cross-reactivity using different serological tests [62]. Therefore, it is imperative to develop new diagnostic tools that allow for a better understanding of the disease.

Given that historical studies [21,22,26], and the findings of the present work, confirmed that TcI is the only genotype detected in Mexico from human isolates from various states of the Mexican Republic, and that there is no genetic variability in any of them, it is possible to propose the development of in silico strategies for the production of antigens to optimise the diagnosis of Chagas disease such as quantitative ELISA methods that use this region of Tc24 without the need for parasitic cultures, and with the possibility of reducing biosafety requirements in diagnostic laboratories. For this reason, the invariance of the Tc24 fragment present in the only DTU (TcI) isolated from human patients in Mexico may offer hope for the production of better diagnostic systems and the potential development of a vaccine.

## Figures and Tables

**Table 1:** Triatomine species infected with *T. cruzi* DTUs in Mexico.

DTU	Vector	State	Reference
TcI			
	*Dipetalogaster maxima*	Baja California Sur	[8,18,19,20]
	*Panstrongylus rufotuberculatus*	Veracruz	[20]
	*Triatoma barberi*	Jalisco, Michoacán, Oaxaca, Puebla, Querétaro	[8,18,21,22,23]
	*Triatoma dimidiata*	Campeche, Chiapas, Michoacán, Quintana Roo, San Luis Potosí, Veracruz, Yucatán	[8,18,20,24,25,26,27,28,29]
	*Triatoma longipennis*	Colima, Jalisco, Michoacán, Nayarit, Zacatecas	[8,21,23,30]
	*Triatoma pallidipennis*	Colima, Jalisco, Michoacán, Morelos, Yucatán,	[8,18,21,23,30]
	*Triatoma picturata*	Jalisco, Nayarit, Sonora	[8,21,26,30]
	*Triatoma phyllosoma*	Nayarit, Zacatecas	[8,21]
	*Triatoma* sp.	Yucatán	[8]
TcII			
	*T. dimidiata*	Veracruz	[20,24,28]
	*T. pallidipennis*	Michoacán	[23]
TcIII			
	*T. dimidiata*	Veracruz	[24]
	*T. pallidipennis*	Michoacán	[23]
TcIV			
	*T. dimidiata*	Quintana Roo, Veracruz	[20,24,29]
	*T. pallidipennis*	Michoacán	[23]
TcV			
	*T. dimidiata*	Veracruz	[24,28]
TcVI			
	*T. dimidiata*	Veracruz	[20,27,28]
Mixed infection			
TcI/TcII			
	*T. dimidiata*	Campeche	[25]
TcI/TcII/TcIV			
	*T. pallidipennis*	Michoacán	[23]

**Table 2:** Mammalian hosts of *T. cruzi* DTUs in Mexico.

DTU	Host/Vector	State	Reference
TcI			
Mammals			
Artiodactyla	*Sus scrofa domesticus*	Campeche	[25]
Carnivora	*Canis lupus familiaris*	Campeche	[25]
Chiroptera	*Artibeus jamaicensis*	Campeche	[25]
	*Carollia brevicauda*	Campeche	[25]
	*Dermanura phaeotis*	Campeche	[25]
	*Sturnira lilium*	Campeche	[25]
	*Sturnira ludovici*	Campeche	[25]
Didelpimorphia	*Didelphis marsupialis*	Yucatán	[8]
	*Didelphis virginiana*	Campeche, Morelos, Veracruz	[8,21,25]
	*Didelphis* sp.	Yucatán	[8,21]
	*Philander opossum*	Veracruz	[8,21]
Rodentia	*Peromyscus yucatanicus*	Campeche	[25]
	*Peromyscus peromyscus*	Puebla	[22]
	*Heteromys gaumeri*	Campeche	[25]
	*Sigmodon toltecus*	Campeche	[25]
	*Mus musculus*	Campeche	[25]
Primates	*Alouatta palliata*	Tabasco	[31]
	*Alouatta pigra*	Tabasco	[31]
	*Ateles geoffroyi*	Veracruz	[31]
	*Homo sapiens*	Guanajuato, Guerrero, Hidalgo, Jalisco, Morelos, Oaxaca, Puebla, San Luis Potosí, Yucatán, Zacatecas	[8,21,22,26,30,32]
TcII			
Artiodactyla	*Ovis aries*	Campeche	[25]
Carnivora	*C. lupus familiaris*	Campeche	[25]
Chiroptera	*Myotis keaysi*	Campeche	[25]
Didelphimorphia	*D. virginiana*	Veracruz	[21]
	*P. oppossum*	Veracruz	[21]
Primates	*A. geoffroyi*	Veracruz	[31]
	*H. sapiens*	Yucatán	[32]
Rodentia	*S. toltecus*	Campeche	[25]
TcV			
Primates	*A. pigra*	Campeche	[31]
TcVI			
Primates	*A. geoffroyi*	Yucatán	[31]
Mixed infection			
TcI/TcII			
Artiodactyla	*O. aries*	Campeche	[25]
	*S. scrofa domesticus*	Campeche	[25]
Carnivora	*C. lupus familiaris*	Campeche	[25]
Chiroptera	*Artibeus lituratus*	Campeche	[25]
Rodentia	*S. toltecus*	Campeche	[25]

**Table 3:** TC24 sequences used in this study.

Sequence ID	DTU	Host	Country	State	Isolate	GenBank Accession Number
009-TCR-NAYA-0I-VE	TcI	Triatominae	Mexico	Nayarit	NAYARIT	OL781152
010-TCR-NI15-0I-HS	TcI	*Homo sapiens*	Mexico	Oaxaca	NINOA 1.5	OL781153
012-TCR-R1H8-0I-HS	TcI	*Homo sapiens*	Mexico	Yucatán	R1H8	OL781155
013-TCR-CALA-0I-VE	TcI	*Triatoma dimidiata*	Mexico	Campeche	CALAKMUL	OL781156
014-TCR-ENTO-0I-VE	TcI	Triatominae	Mexico	Morelos	ENTO	OL781157
015-TCR-INC1-0I-HS	TcI	*Homo sapiens*	Mexico	Oaxaca	INC-1	OL781158
016-TCR-INC5-0I-HS	TcI	*Homo sapiens*	Mexico	Guanajuato	INC-5	OL781159
017-TCR-INC6-0I-HS	TcI	*Homo sapiens*	Mexico	Oaxaca	INC-6	OL781160
018-TCR-INC9-0I-HS	TcI	*Homo sapiens*	Mexico	Guerrero	INC-9	OL781161
019-TCR-IN10-01-HS	TcI	*Homo sapiens*	Mexico	Guanajuato	INC-10	OL781162
020-TCR-CAM7-0I-VE	TcI	*Triatoma dimidiata*	Mexico	Campeche	CAM-7	OL781163
021-TCR-CAM8-0I-VE	TcI	*Triatoma dimidiata*	Mexico	Campeche	CAM-8	OL781164
022-TCR-HUEY-0I-VE	TcI	*Triatoma barberi*	Mexico	Estado de México	HUEYPOXTLA	OL781165
023-TCR-NINO-0I-HS	TcI	*Homo sapiens*	Mexico	Oaxaca	NINOA	OL781166
024-TCR-SONT-0I-TD	TcI	*Triatoma dimidiata*	Mexico	Veracruz	SONTECOMAPAN	OL781167
025-TCR-TEZO-0I-VE	TcI	*Triatoma barberi*	Mexico	Estado de México	TEZONTLALPAN	OL781168
6Morelos	TcI	*Homo sapiens*	Mexico	Morelos	MORELOS	OL781175
7CoculaJALISCO	TcI	Triatominae	Mexico	Jalisco	COCULA	OL781176
9JerboYUCATAN	TcI	*Homo sapiens*	Mexico	Yucatán	JERBO	OL781177
13Zacatecas	TcI	*Homo sapiens*	Mexico	Zacatecas	ZACATECAS	OL781178
14PeNaGUERRERO	TcI	*Homo sapiens*	Mexico	Guerrero	PEÑA	OL781179
15PetaquillasGUERRER	TcI	Triatominae	Mexico	Guerrero	PETAQUILLAS	OL781180
23ProgresoMOLRELOS	TcI	Triatominae	Mexico	Morelos	PROGRESO	OL781181
25Yuc	TcI	Triatominae	Mexico	Yucatán	YUC	OL781182
ENTOMO	TcI	ND	ND	ND	ENTOMO	OL781183
011-TCR-DM22-0I-DM	TcI	*Didelphis marsupialis*	Brazil	ND	DM28	OL781154
035-DTUI	TcI	*Didelphis marsupialis*	Venezuela	ND	DM28	OL781169
036-DTUII	TcII	*Homo sapiens*	Brazil	ND	000Y	OL781170
037-DTUIII	TcIII	*Panstrongylus geniculatus*	Brazil	Manaus	3663	OL781171
038-DTUIV	TcIV	*Rhodnius brethesi*	Brazil	Barcelos	4167	OL781172
039-DTUV	TcV	*Triatoma infestans*	Argentina	Chaco	LL014	OL781173
040-DTUVI	TcVI	*Triatoma infestans*	Brazil	Rio Grande do Sul	CL Brener	OL781174
002-TCR-MAST-NR-CL	ND	*Canis lupus familiaris*	US	Texas	MASTIFF	DQ183066
004-TCR-CLBR-VI-HS	TcVI	*Triatoma infestans*	Brazil	ND	CLBRENER	XM_800482.1
005-TCR-000Y-II-HS	TcII	*Homo sapiens*	Brazil	ND	000Y	D87512.1
006-TCR-00Y2-II-HS	TcII	*Homo sapiens*	Brazil	ND	000Y	S43664.1
007-TCR-CLB2-VI-HS	TcVI	*Triatoma infestans*	Brazil	ND	CL BRENER	AF192980.2
001-TCO-025E-NR-RR	*Trypanosoma conorhini*	*Rattus rattus*	Brazil	NR	025E	XM_029375354.1

## Data Availability

The data that support the results of this study are available in GenBank, under the following accessions numbers: OL781152-OL781183.

## References

[1] World Health Organization (2015). Chagas disease in Latin America: An epidemiological update based on 2010 estimates. Wkly. Epidemiol. Rec..

[2] Rassi A.J., Rassi A., Marin-Neto J. (2010). Chagas disease. Lancet.

[3] Lee B., Bacon K., Bottazzi M., Hotez P. (2013). Global economic burden of Chagas disease: A computational simulation model. Lancet Infect. Dis..

[4] Requena-Méndez A., Albajar-Viñas P., Angheben A., Chiodini P., Gascón J., Muñoz J. (2014). Health policies to control Chagas disease transmission in European countries. PLoS Negl. Trop. Dis..

[5] Angheben A., Boix L., Buonfrate D., Gobbi F., Bisoffi Z., Pupella S., Gandini G., Aprili G. (2015). Chagas disease and transfusion medicine: A perspective from non-endemic countries. Blood Transfus..

[6] Ramírez J.D., Hernández C., Montilla M., Zambrano P., Flórez A.C., Parra E., Cucunubá Z.M. (2014). First report of human *Trypanosoma cruzi* infection attributed to TcBat genotype. Zoonoses Public Health.

[7] Zingales B., Andrade S., Briones M., Campbell D., Chiari E., Fernandes O., Guhl F., Lages-Silva E., Macedo A.M., Machado C.R. (2009). A new consensus for *Trypanosoma cruzi* intraspecific nomenclature: Second revision meeting recommends TcI to TcVI. Mem. Inst. Oswaldo. Cruz..

[8] Brenière S., Waleckx E., Barnabé C. (2016). Over six thousand *Trypanosoma cruzi* strains classified into Discrete Typing Units (DTUs): Attempt at an inventory. PLoS Negl. Trop. Dis..

[9] Centro Nacional de Vigilancia Epidemiológica y Control de Enfermedades (CENAVECE) (2022). Anuarios de Morbilidad Durante el Periodo 1995–2010. Anuarios de Morbilidad, 1984–2017.

[10] SSA—Secretaría de Salud (2014). Norma Oficial Mexicana NOM-032-SSA2-2014, Para la Vigilancia Epidemiológica, Prevención y Control de Enfermedades Transmitidas por Vector.

[11] Ibarra-Cerdeña C., Sánchez-Cordero V., Townsend P.A., Ramsey J. (2009). Ecology of North American Triatominae. Acta Trop..

[12] Magallón-Gastélum E., Lozano-Kasten F., Gutierréz M., Flores-Pérez A., Sánchez B., Espinoza B., Bosseno M., Brenière S. (2006). Epidemiological risk for *Trypanosoma cruzi* transmission by species of *Phyllosoma* complex in the occidental part of Mexico. Acta Trop..

[13] Sandoval-Ruiz C., Cervantes-Peredo L., Mendoza-Palmero F., Ibáñez-Bernal S. (2012). The Triatominae (Hemiptera: Heteroptera: Reduviidae) of Veracruz, Mexico: Geographic distribution, taxonomic redescriptions, and a key. Zootaxa.

[14] Arce-Fonseca M., Carrillo-Sánchez S.C., Molina-Barrios R.M., Martínez-Cruz M., Cedillo-Cobián J.R., Henao-Díaz Y.A., Rodríguez-Morales O. (2017). Seropositivity for *Trypanosoma cruzi* in domestic dogs from Sonora, Mexico. Infect. Dis. Poverty.

[15] Buekens P., Cafferata M., Alger J., Althabe F., Belizán J., Carlier Y., Ciganda A., Del Cid J.H., Dumonteil E., Gamboa-León R. (2018). Congenital transmission of *Trypanosoma cruzi* in Argentina, Honduras, and Mexico: An Observational Prospective Study. Am. J. Trop. Med. Hyg..

[16] Montes-Rincón L., Galaviz-Silva L., Molina-Garza Z. (2018). Anticuerpos anti-*Trypanosoma cruzi* en migrantes latinoamericanos en tránsito por el cruce fronterizo entre México y los Estados Unidos. Biomédica.

[17] Newton-Sánchez O., Espinoza-Gómez F., Melnikov V., Delgado-Enciso I., Rojas-Larios F., Dumonteil E., Trujillo-Hernández B., De La Cruz-Ruíz M. (2017). Seroprevalence of *Trypanosoma cruzi* (TC) and risk factors in Colima, Mexico. Gac. Med. Mex..

[18] Zumaya-Estrada F., Messenger L., Lopez-Ordonez T., Lewis M., Flores-Lopez C., Martínez-Ibarra A., Pennington P.M., Cordon-Rosales C., Carrasco H.V., Segovia M. (2012). North American import? Charting the origins of an enigmatic *Trypanosoma cruzi* domestic genotype. Parasit. Vectors.

[19] Rivas-García L., Carballo-Amador M., Flores-López C. (2020). Design of a AFLP-PCR and PCR-RFLP test that identify the majority of discrete typing units of *Trypanosoma cruzi*. PLoS ONE.

[20] Díaz-Valdez J., Martínez I., Rodríguez-Moreno Á., Gutiérrez-Granados G., León-Villegas R., Sánchez-Cordero V., Fraga-Nodarse J., Ángles-Chimal J., Espinoza E. (2021). Multiple discrete typing units of *Trypanosoma cruzi* infect sylvatic *Triatoma dimidiata* and *Panstrongylus rufotuberculatus* in Southeast Mexico. Am. J. Trop. Med. Hyg..

[21] Bosseno M., Barnabé C., Magallón-Gastélum E., Lozano-Kasten F., Ramsey J., Espinoza B., Brenière S. (2002). Predominance of *Trypanosoma cruzi* lineage I in Mexico. J. Clin. Microbiol..

[22] Sánchez-Guillén M., Bernabé C., Tibayrenc M., Zavala-Castro J., Totolhua J., Méndez-López J., González-Mejía M.E., Torres-Rasgado E., López-Colombo A., Pérez-Fuentes R. (2006). *Trypanosoma cruzi* strains isolated from human, vector, and animal reservoir in the same endemic region in Mexico and typed as *T. cruzi I*, discrete typing unit 1 exhibit considerable biological diversity. Mem. Inst. Oswaldo. Cruz..

[23] Ibáñez-Cervantes G., Martínez-Ibarra A., Nogueda-Torres B., López-Orduña E., Alonso A., Perea C., Maldonado T., Hernández J.M., León-Avila G. (2013). Identification by Q-PCR of *Trypanosoma cruzi* lineage and determination of blood meal sources in triatomine gut samples in México. Parasitol. Int..

[24] Ramos-Ligonio A., Torres-Montero J., López-Monteon A., Dumonteil E. (2012). Extensive diversity of *Trypanosoma cruzi* discrete typing units circulating in *Triatoma dimidiata* from central Veracruz, Mexico. Infect. Genet. Evol..

[25] López-Cancino S., Tun-Ku E., De la Cruz-Felix H.K., Ibarra-Cerdeña C., Izeta-Alberdi A., Pech-May A., Mazariegos-Hidalgo C., Valdez-Tah A., Ramsey J. (2015). Landscape ecology of *Trypanosoma cruzi* in the southern Yucatan Peninsula. Acta Trop..

[26] Monteón V., Triana-Chávez O., Mejía-Jaramillo A., Pennignton P., Ramos-Ligonio Á., Acosta K., Lopez R. (2016). Circulation of Tc Ia discrete type unit *Trypanosoma cruzi* in Yucatan Mexico. J. Parasit. Dis..

[27] Pech-May A., Mazariegos-Hidalgo C., Izeta-Alberdi A., López-Cancino S., Tun-Ku E., De la Cruz-Félix K., Ibarra-Cerdeña C., Ittig R.G., Ramsey J. (2019). Genetic variation and phylogeography of the *Triatoma dimidiata* complex evidence a potential center of origin and recent divergence of haplogroups having differential *Trypanosoma cruzi* and DTU infections. PLoS Negl. Trop. Dis..

[28] Murillo-Solano C., Ramos-Ligonio A., López-Monteon A., Guzmán-Gómez D., Torres-Montero J., Herrera C., Dumonteil E. (2021). Diversity of *Trypanosoma cruzi* parasites infecting *Triatoma dimidiata* in Central Veracruz, Mexico, and their One Health ecological interactions. Infect. Genet. Evol..

[29] Dorn P., McClure A., Gallaspy M., Waleckx E., Woods A., Monroy M., Stevens L. (2017). The diversity of the Chagas parasite, *Trypanosoma cruzi*, infecting the main Central American vector, *Triatoma dimidiata*, from Mexico to Colombia. PLoS Negl. Trop. Dis..

[30] Gómez-Hernández C., Pérez S., Rezende-Oliveira K., Barbosa C., Lages-Silva E., Ramírez L., Ramírez J. (2019). Evaluation of the multispecies coalescent method to explore intra- *Trypanosoma cruzi I* relationships and genetic diversity. Parasitology.

[31] Rovirosa-Hernández M., López-Monteon A., García-Orduña F., Torres-Montero J., Guzmán-Gómez D., Dumonteil E., Waleckx E., Lagunes-Merino O., Canales-Espinoza D., Ramos-Ligonio A. (2021). Natural infection with *Trypanosoma cruzi* in three species of non-human primates in southeastern Mexico: A contribution to reservoir knowledge. Acta Trop..

[32] Herrera C., Truyens C., Dumonteil E., Alger J., Sosa-Estani S., Cafferata M.L., Gibbons L., Ciganda A., Matute M.L., Zuniga C. (2019). Phylogenetic analysis of *Trypanosoma cruzi* from pregnant women and newborns from Argentina, Honduras, and Mexico suggests an association of parasite haplotypes with congenital transmission of the parasite. J. Mol. Diagn..

[33] Rawal K., Sinha R., Abbasi B.A., Chaudhary A., Nath S.K., Kumari P., Preeti P., Saraf D., Singh S., Mishra K. (2021). Identification of vaccine targets in pathogens and design of a vaccine using computational approaches. Sci. Rep..

[34] Limon-Flores A., Cervera-Cetina R., Tzec-Arjona J., Ek-Macias L., Sánchez-Burgos G., Ramirez-Sierra M., Cruz-Chan V., VanWynsberghe N.R., Dumonteil E. (2010). Effect of a combination DNA vaccine for the prevention and therapy of *Trypanosoma cruzi* infection in mice: Role of CD4+ and CD8+ T cells. Vaccine.

[35] Hotez P.J., Dumonteil E., Heffernan M.J., Bottazzi M.E. (2013). Innovation for the `Bottom 100 Million’: Eliminating Neglected Tropical Diseases in the Americas. Adv. Exp. Med. Biol..

[36] Dumonteil E., Bottazzi M., Zhan B., Heffernan M., Jones K., Valenzuela J., Kamhawi S., Ortega J., Ponce de Leon S.R., Lee B.Y. (2012). Accelerating the development of a therapeutic vaccine for human Chagas disease: Rationale and prospects. Expert. Rev. Vaccines.

[37] Arnal A., Villanueva-Lizama L., Teh-Poot C., Herrera C., Dumonteil E. (2020). Extent of polymorphism and selection pressure on the *Trypanosoma cruzi* vaccine candidate antigen Tc24. Evol. Appl..

[38] Espinoza B., Rico T., Sosa S., Oaxaca E., Vizcaino-Castillo A., Caballero M., Martínez I. (2010). Mexican *Trypanosoma cruzi T. cruzi I* strains with different degrees of virulence induce diverse humoral and cellular immune responses in a murine experimental infection model. J. Biomed. Biotechnol..

[39] López-Olmos V., Pérez-Nasser N., Piñero D., Ortega E., Hernandez R., Espinoza B. (1998). Biological characterization and genetic diversity of Mexican isolates of *Trypanosoma cruzi*. Acta Trop..

[40] Da Cruz Moreira O., Ramirez J.C. (2019). Genotyping of *Trypanosoma cruzi* from Clinical Samples by Multilocus Conventional PCR. Methods Mol. Biol..

[41] Colunga-Salas P., Hernández-Canchola G. (2021). Bats and humans during the SARS-CoV-2 outbreak: The case of bat-coronaviruses from Mexico. Transbound. Emerg. Dis..

[42] Sánchez-Montes S., Salceda-Sánchez B., Bermúdez S., Aguilar-Tipacamú G., Ballados-González G., Huerta H., Aguilar-Domínguez M., Delgado-de la Mora J., Licona-Enríquez J.D., Delgado-de la Mora D. (2021). *Rhipicephalus sanguineus* complex in the Americas: Systematic, genetic diversity, and geographic insights. Pathogens.

[43] Nguyen L., Schmidt H., Von Haeseler A., Minh B. (2015). IQ-TREE: A fast and effective stochastic algorithm for estimating Maximum-Likelihood phylogenies. Mol. Biol. Evol..

[44] Kalyaanamoorthy S., Minh B., Wong T., Von Haeseler A., Jermiin L. (2017). ModelFinder: Fast model selection for accurate phylogenetic estimates. Nat. Methods.

[45] Ronquist F., Teslenko M., Van Der Mark P., Ayres D., Darling A., Höhna S., Larget B., Liu L., Suchard M.A., Huelsenbeck J.P. (2012). MrBayes 3.2: Efficient Bayesian phylogenetic inference and model choice across a large model space. Syst. Biol..

[46] Lanfear R., Frandsen P., Wright A., Senfeld T., Calcott B. (2017). PartitionFinder 2: New methods for selecting partitioned models of evolution for molecular and morphological phylogenetic analyses. Mol. Biol. Evol..

[47] Guindon S., Dufayard J., Lefort V., Anisimova M., Hordijk W., Gascuel O. (2010). New algorithms and methods to estimate Maximum-Likelihood phylogenies: Assessing the performance of PhyML 3.0. Syst. Biol..

[48] Rambaut A., Drummond A., Xie D., Baele G., Suchard M. (2018). Posterior summarization in Bayesian phylogenetics using Tracer 1.7. Syst. Biol..

[49] Librado P., Rozas J. (2009). DnaSP v5: A software for comprehensive analysis of DNA polymorphism data. Bioinformatics.

[50] Jombart T. (2008). adegenet: A R package for the multivariate analysis of genetic markers. Bioinformatics.

[51] Mohamed N.S., AbdElbagi H., Elsadig A.R., Ahmed A.E., Mohammed O.Y., Elssir L.T., Elnour M.A., Ali Y., Ali M.S., Altahir O. (2021). Assessment of genetic diversity of *Plasmodium falciparum* circumsporozoite protein in Sudan: The RTS,S leading malaria vaccine candidate. Malar. J..

[52] Flores-Alanis A., González-Cerón L., Santillán-Valenzuela F., Ximenez C., Sandoval-Bautista M.A., Cerritos R. (2022). Spatiotemporal Changes in *Plasmodium vivax* msp142 Haplotypes in Southern Mexico: From the Control to the Pre-Elimination Phase. Microorganisms.

[53] Dzul-Huchim V.M., Ramirez-Sierra M.J., Martinez-Vega P.P., Rosado-Vallado M.E., Arana-Argaez V.E., Ortega-Lopez J., Gusovsky F., Dumonteil E., Cruz-Chan J.V., Hotez P. (2022). Vaccine-linked chemotherapy with a low dose of benznidazole plus a bivalent recombinant protein vaccine prevents the development of cardiac fibrosis caused by *Trypanosoma cruzi* in chronically-infected BALB/c mice. PLoS Negl. Trop. Dis..

[54] González-López C., Chen W.H., Alfaro-Chacón A., Villanueva-Lizama L.E., Rosado-Vallado M., Ramirez-Sierra M.J., Teh-Poot C.F., Pollet J., Asojo O., Jones K.M. (2022). A novel multi-epitope recombinant protein elicits an antigen-specific CD8+ T cells response in *Trypanosoma cruzi*-infected mice. Vaccine.

[55] Quijano-Hernández I.A., Castro-Barcena A., Vázquez-Chagoyán J.C., Bolio-González M.E., Ortega-López J., Dumonteil E. (2013). Preventive and therapeutic DNA vaccination partially protect dogs against an infectious challenge with *Trypanosoma cruzi*. Vaccine.

[56] Dumonteil E., Herrera C., Marx P.A. (2022). Safety and preservation of cardiac function following therapeutic vaccination against *Trypanosoma cruzi* in rhesus macaques. J. Microbiol. Immunol. Infect..

[57] Viettri M., Lares M., Medina M., Herrera L., Ferrer E. (2022). Evaluation of commercial kits for the immunological and molecular diagnosis of Chagas disease in endemic areas of Venezuela. Enferm. Infecc. Microbiol. Clin..

[58] Chakravarti I., Miranda-Schaeubinger M., Ruiz-Remigio A., Briones-Garduño C., Fernández-Figueroa E.A., Villanueva-Cabello C.C., Borge-Villareal A., Bejar-Ramírez Y., Pérez-González A., Rivera-Benitez C. (2022). Chagas Disease in Pregnant Women from Endemic Regions Attending the Hospital General de Mexico, Mexico City. Trop. Med. Infect. Dis..

[59] Mita-Mendoza N.K., McMahon E., Kenneson A., Barbachano-Guerrero A., Beltran-Ayala E., Cueva C., King C.A., Lupone C.D., Castro-Sesquen Y.E., Gilman R.H. (2018). Chagas Disease in Southern Coastal Ecuador: Coinfections with Arboviruses and a Comparison of Serological Assays for Chagas Disease Diagnosis. Am. J. Trop. Med. Hyg..

[60] Llano M., Pavía P., Flórez A.C., Cuéllar A., González J.M., Puerta C. (2014). Evaluación preliminar de la prueba comercial Chagas (*Trypanosoma cruzi*) IgG-ELISA ® en individuos colombianos [Preliminary evaluation of the commercial kit Chagas (*Trypanosoma cruzi*) IgG-ELISA ® in Colombian individuals]. Biomedica.

[61] Luquetti A.O., Espinoza B., Martínez I., Hernández-Becerril N., Ponce C., Ponce E., Reyes P.A., Hernández O., López R., Monteón V. (2009). Performance levels of four Latin American laboratories for the serodiagnosis of Chagas disease in Mexican sera samples. Mem. Inst. Oswaldo Cruz.

[62] Vexenat A.D.C., Santana J.M., Teixeira A.R. (1996). Cross-reactivity of antibodies in human infections by the kinetoplastid protozoa *Trypanosoma cruzi*, *Leishmania chagasi* and *Leishmania* (*viannia*) *braziliensis*. Rev. Inst. Med. Trop. Sao Paulo.

